# Therapeutic effects of anti‐GM2 CAR‐T cells expressing IL‐7 and CCL19 for GM2‐positive solid cancer in xenograft model

**DOI:** 10.1002/cam4.5907

**Published:** 2023-04-09

**Authors:** Takahiro Sasaki, Yukimi Sakoda, Keishi Adachi, Yoshihiro Tokunaga, Koji Tamada

**Affiliations:** ^1^ Department of Immunology Yamaguchi University Graduate School of Medicine Ube Japan; ^2^ Department of Endocrinology, Metabolism, Hematological Science and Therapeutics Yamaguchi University Graduate School of Medicine Ube Japan

**Keywords:** CAR‐T cell, chemokine, cytokine, ganglioside, solid cancers

## Abstract

**Background:**

While chimeric antigen receptor (CAR)‐T cell therapy has demonstrated excellent efficacy in hematopoietic malignancies, its clinical application in solid cancers has yet to be achieved. One of the reasons for such hurdle is a lack of suitable CAR targets in solid cancers.

**Methods:**

GM2 is one of the gangliosides, a group of glycosphingolipids with sialic acid in the glycan, and overexpressed in various types of solid cancers. In this study, by using interleukin (IL)‐7 and chemokine (C‐C motif) ligand 19 (CCL19)‐producing human CAR‐T system which we previously developed, a possibility of GM2 as a solid tumor target for CAR‐T cell therapy was explored in a mouse model with human small‐cell lung cancer.

**Results:**

Treatment with anti‐GM2 IL‐7/CCL19‐producing CAR‐T cells induced complete tumor regression along with an abundant T cell infiltration into the solid tumor tissue and long‐term memory responses, without any detectable adverse events. In addition, as measures to control cytokine‐release syndrome and neurotoxicity which could occur in association with clinical use of CAR‐T cells, we incorporated Herpes simplex virus‐thymidine kinase (HSV‐TK), a suicide system to trigger apoptosis by administration of ganciclovir (GCV). HSV‐TK‐expressing anti‐GM2 IL‐7/CCL19‐producing human CAR‐T cells were efficiently eliminated by GCV administration in vivo.

**Conclusions:**

Our study revealed the promising therapeutic efficacy of anti‐GM2 IL‐7/CCL19‐producing human CAR‐T cells with an enhanced safety for clinical application in the treatment of patients with GM2‐positive solid cancers.

## Graphical abstract

Administration of anti‐GM2 IL‐7/CCL19‐producing chimeric antigen receptor (CAR)‐T cells induced complete regression of xenografted human small‐cell lung cancer. Abundant T cell infiltration into the solid cancer tissue and long‐term memory responses was induced along with antitumor efficacy.

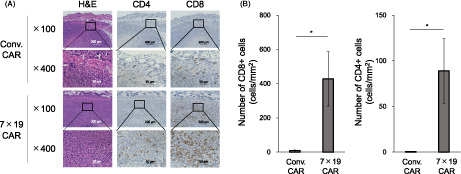

## 1 INTRODUCTION

Anti‐CD19 and anti‐BCMA chimeric antigen receptor (CAR)‐T cells have demonstrated significant therapeutic effects on hematological B‐cell malignancies and multiple myeloma, respectively, and thus have been clinically approved.^1^, ^2^, ^3^, ^4^, ^5^ On the other hand, the efficacy of CAR‐T cell therapy for solid cancers has yet to be established. Since the number of solid cancer patients occupies over 90% of all cancer patients, the development of next‐generation technologies to make CAR‐T cells effective for solid cancers is highly demanded. Insufficient efficacy of CAR‐T cells could be attributed to several features of solid cancers different from those of hematological malignancies. First, due to the heterogeneous nature of solid cancers, it is difficult to identify a target that is selectively expressed on majority of cancer cells but not on normal cells.^6^, ^7^, ^8^ Second, solid cancers establish immunological barriers caused by various immunosuppressive mechanisms in the tumor microenvironment, by which immune effector cells including CAR‐T cells hardly demonstrate optimal functions.^6^, ^7^, ^8^ In addition, solid cancers also generate physiological barriers such as fibrotic stroma, which inhibit the accumulation and infiltration of immune cells inside cancer tissues.^6^, ^7^, ^8^

To overcome these barriers, various technological improvements in CAR‐T cells have been reported.^6^, ^7^, ^8^ Recently, our group developed the modified CAR‐T cells that concomitantly express interleukin‐7 (IL‐7) and chemokine (C‐C motif) ligand 19 (CCL19) (hereafter referred as 7 × 19 CAR‐T cells), so as to induce an active migration of T cells and dendritic cells (DC) by the effects of CCL19 and enhance T cell proliferation and memory formation by the effects of IL‐7.^9^, ^10^ The antitumor effects of 7 × 19 CAR‐T cells were significantly superior to those of conventional CAR‐T cells in various solid cancer models using syngeneic mouse‐derived tumors as well as patient‐derived xenograft tumors.^9^, ^10^ The potent efficacy of 7 × 19 CAR‐T cells is dependent, at least in part, on endogenous non‐CAR‐T cells,^9^, ^10^ indicating a unique mechanism different from other armored CAR technologies. Genetic modification of tumor antigen‐specific T cell receptor (TCR)‐T cells to produce IL‐7 and CCL19 also significantly enhanced the therapeutic efficacy in mouse models.^11^ These findings together suggest that concomitant expression of IL‐7 and CCL19 can be a platform technology to boost the therapeutic potential of the effector T cell therapies against solid cancers. To consolidate this concept, further studies using different target molecules are needed.

GM2 is one of the gangliosides, which is a group of glycosphingolipids containing sialic acid in the glycan.^12^ More than 200 types of gangliosides have been discovered and are known to exist in cell membranes and play important roles in membrane formation, neuronal differentiation, cell adhesion, and signal transduction.^12^, ^13^ Gangliosides are abundant in nervous tissue where GM1, GD1a, GD1b, GT1b, and GQ1b account for most of them, while other gangliosides including GM2 are minor components in normal nervous systems.^12^, ^13^ Interestingly, GM2 is overexpressed in various cancer tissues including lung cancer, colon cancer, ovarian cancer, and malignant pleural mesothelioma,^14^, ^15^, ^16^, ^17^ suggesting that GM2 can be a potential target for cancer immunotherapy. Monoclonal antibody (mAb) against GM2 with enhanced activity of antibody‐dependent cellular cytotoxicity (ADCC) was developed and examined in Phase I/II clinical trials, which resulted in insufficient efficacy while the safety was confirmed.^18^ Besides GM2, an example of cancer immunotherapy targeting ganglioside is anti‐GD2 CAR‐T cells for neuroblastoma and glioma.^19^, ^20^ In clinical trials, anti‐GD2 CAR‐T cells induced therapeutic benefits in some cases, while no obvious neurotoxicity was observed.^19^, ^20^ From these lines of evidence, we anticipated that GM2 can be a target of novel CAR‐T cell therapy for the treatment of various solid cancers.

In order to explore the IL‐7/CCL19‐producing system as a platform technology to enhance the therapeutic efficacy of CAR‐T cells and to address the possibility of GM2 as a CAR target in solid cancers, we generated anti‐GM2 7 × 19 CAR‐T cells in this study and examined its efficacy in immunodeficient mouse model with human small‐cell lung cancer (SCLC). In addition, the induction of target‐specific long‐term memory responses of anti‐GM2 7 × 19 CAR‐T cells was addressed. Furthermore, we examined the effect of Herpes simplex virus‐thymidine kinase (HSV‐TK), which was installed in anti‐GM2 7 × 19 CAR construct as a suicide switch to eliminate CAR‐T cells in response to the administration of ganciclovir (GCV).

## 2 MATERIALS AND METHODS

### 2.1 Mice and Cell lines

Female NOD.Cg‐*Prkdc* ^*scid*^ *Il2rg* ^*tm1Sug*^ *B2m* ^*em1Tac*^ *H2‐Ab1* ^*tm1Doi*^/Jic (NOG‐ΔMHC) mice at 7–8 weeks old were purchased from CLEA Japan and used in the all in vivo experiments. The mice were maintained under specific pathogen‐free conditions in our facility as previously reported,^10^ and given enrofloxacin orally for a week after arrival or until the termination of experiments. All animal experiments were approved by the Institutional Animal Care and Use Committee at Yamaguchi University. Lu‐135, a human SCLC cell line, KMS11, a human myeloma cell lime, and A549‐Luc, a human lung adenocarcinoma cell line that stably expresses luciferase, were purchased from the Japanese Collection of Research Bioresources Cell Bank. NCI‐H2052, a human mesothelioma cell line, and SW480, a human colon adenocarcinoma cell line, were purchased from ATCC. Lu‐135 cells were cultured in an RPMI‐1640 (Gibco) medium supplemented with 10% heat‐inactivated fetal bovine serum (FBS; Gemini BioProducts), and 1% penicillin–streptomycin sulfate (Wako). KMS11 and NCI‐H2052 cells were cultured in RPMI‐1640 medium supplemented with 10% heat‐inactivated FBS, 1% penicillin–streptomycin sulfate, 25 mM HEPES (Sigma‐Aldrich), and 50 mM 2‐mercaptoethanol (Thermo Fisher Scientific). A549‐Luc cells and SW480 cells were cultured in Dulbecco's modified Eagle's medium (Gibco) supplemented with 10% heat‐inactivated FBS and 1% penicillin–streptomycin sulfate.

### 2.2 Design of CAR‐expressing vectors and gene transfer to human T cells

Anti‐GM2 single‐chain variable fragment (scFv) was designed with variable region sequences of heavy and light chains derived from humanized anti‐GM2 mAb.^16^, ^17^, ^18^ CAR construct was designed with the scFv, the transmembrane sequence of human CD8α, cytoplasmic sequences of human CD28, 4‐1BB, and CD3ζ, and cloned into the retroviral pMSGV1 vector.^21^, ^22^ Human IL‐7, CCL19, and HSV‐TK were expressed in addition to CAR by using self‐cleavable 2A linear sequence. Gene transfer to human T cells was conducted as previously described.^10^, ^22^ In some experiments, retroviruses were produced by the producer cell line which was stably transduced with the CAR‐expressing gene together with the envelope gene. The transduction efficiency of CAR was assessed by flow cytometry using anti‐idiotype mAb against GM2 CAR. The production of human IL‐7 and CCL19 in the culture supernatants was measured as previously reported.^10^ Un‐transduced activated T cells were generated by the same culture conditions without retrovirus infection.

### 2.3 Flow cytometry

Unconjugated humanized anti‐GM2 mAb (Creative Biolabs) and secondary APC‐conjugated anti‐human IgG Fc mAb (clone HP6017; BioLegend) were used to detect the surface GM2. The CAR‐expressing T cells were detected with APC‐Cy7‐conjugated anti‐CD3 mAb (clone HIT3a; BioLegend), PerCPCy5.5‐conjugated anti‐CD4 mAb (clone OKT4; BioLegend), APC‐conjugated anti‐human CD8 (clone HIT8a; BioLegend), and/or biotinylated anti‐idiotype Ab against anti‐GM2 CAR (Cell Engineering Corporation), followed by PE‐conjugated streptavidin (BioLegend). Zombie Yellow viability dye (BioLegend) and PE‐conjugated anti‐CD45 mAb (clone HI30; BioLegend) were used to detect viable immune and non‐immune cells after in vitro co‐culture assay. Human TruStain FcX (BioLegend) was used to block nonspecific binding of mAb with Fcγ receptors. Flow cytometric data were acquired and analyzed as previously reported.^10^

### 2.4 In vitro assay of cytotoxicity

To assess a cytotoxic activity in vitro, co‐culture of CAR‐T or un‐transduced T cells (1 × 10^5^ cells/well) with tumor cells, and subsequent analysis of the remaining tumor cells and T cells, as well as interferon (IFN)‐γ production, were conducted as previously reported.^10^ The percentage of CAR‐positive cells, which varied among the CAR constructs, was adjusted to the same level by adding un‐transduced T cells prior to the co‐culture.

### 2.5 In vivo antitumor mouse model

NOG‐ΔMHC mice were inoculated subcutaneously (s.c.) with 5 × 10^6^ or 1 × 10^7^ Lu‐135 tumor cells on the right flank on day 0. Three days later, 1 × 10^7^ CAR‐T or un‐transduced T cells were injected intravenously (i.v.) through the tail vein. The percentage of CAR‐positive cells, which varied among the CAR constructs, was adjusted to the same level by adding un‐transduced T cells prior to the injection. In tumor rechallenge experiments, NOG‐ΔMHC mice that had previously rejected Lu‐135 tumor by the treatment with 7 × 19 CAR‐T cells were inoculated s.c. with 1 × 10^7^ Lu‐135 or 3 × 10^6^ KMS11 on the right flank and 5 × 10^6^ SW480 cells on the left flank of the mice, respectively. In all in vivo experiments, tumor size was assessed as previously reported,^10^ and the mice were euthanized when the tumor volume exceeded 1500 mm^3^.

### 2.6 Histopathological analysis

To analyze T cell infiltration in the tumor tissues, the tumor mass was resected 9 days after i.v. administration of CAR‐T cells, fixed with 10% formaldehyde, and then embedded with paraffin. The tissue sections were analyzed by H&E staining or immunohistochemical (IHC) staining with rabbit anti‐CD4 mAb (clone SP35; Roche), rabbit anti‐CD8 mAb (clone SP57; Roche), rabbit anti‐Granzyme B polyclonal Ab (Roche) and mouse anti‐PD‐1 mAb (clone NAT105; Roche). Microscopic analyses for H&E and IHC samples were conducted using a BZ‐X710 fluorescence microscope and BZ‐X analyzer (KEYENCE).

### 2.7 Analysis of HSV‐TK suicide activity

For in vitro analysis of HSV‐TK activity, CAR‐T cells with or without HSV‐TK expression were cultured in the presence of GCV (Mitsubishi Tanabe Pharma Corporation) at a concentration of 0, 0.1, 1, 10 μM. Three days later, the number of CAR‐T cells was analyzed by flow cytometry. For in vivo analysis of HSV‐TK activity, NOG‐ΔMHC mice were inoculated s.c. with 1 × 10^7^ Lu‐135 tumor cells on the right flank on day 0, followed by i.v. injection of 1 × 10^7^ HSV‐TK‐expressing 7 × 19 CAR‐T cells on day 3. The mice were injected intraperitoneally (i.p.) with GCV (100 mg/kg) on days 7 and 21, and the proportion of CAR‐T cells in peripheral blood mononuclear cells (PBMC) was analyzed by flow cytometry on days 5, 11, 18, and 25. The tumor size was also measured twice a week with a digital caliper.

### 2.8 Statistics

Statistical analyses of in vitro experiments and in vivo mouse survival assays were examined by two‐sided Student's t‐tests and Log‐rank test, respectively. *p* < 0.05 was considered statistically significant.

## 3 RESULTS

### 3.1 Generation of anti‐GM2 CAR‐T cells expressing IL‐7, CCL19 and HSV‐TK

**FIGURE 1:**
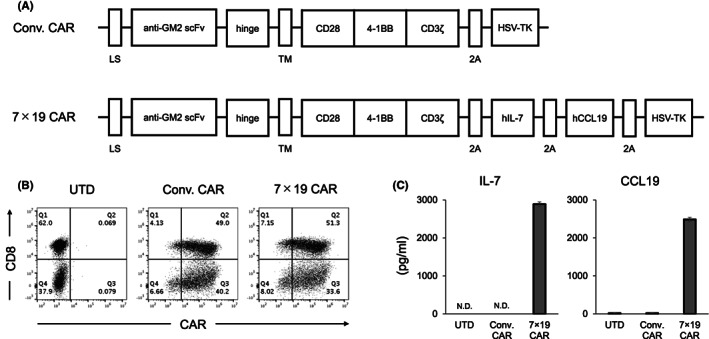
Generation and characterization of anti‐GM2 CAR‐T cells expressing IL‐7, CCL19, and herpes simplex virus‐thymidine kinase. (A) Schematic representation of Conv. CAR and 7 × 19 CAR against GM2. LS; leader sequence, TM; transmembrane region. (B) Human PBMC transduced with Conv. CAR or 7 × 19 CAR were stained with anti‐idiotype Ab against anti‐GM2 scFv to detect CAR expression, along with anti‐CD8 Ab. UTD T cells were examined as a negative control. The percentage of cells in each quadrant is indicated. (C) The culture supernatants from Conv. CAR‐T and 7 × 19 CAR‐T cells were harvested 4 days after gene transduction, and the concentrations of IL‐7 and CCL19 were measured by ELISA. As a control, the culture supernatant obtained from UTD T cells at the same time point was examined. Data are shown as mean ± standard error of triplicate samples. N.D., not detected.

To evaluate the therapeutic potential of anti‐GM2 CAR‐T cells against GM2‐positive solid cancers, we first designed a third‐generation CAR construct containing anti‐GM2 scFv, hinge and transmembrane region, CD28, 4‐1BB, and CD3ζ signaling motifs (hereafter referred to as Conv. CAR). HSV‐TK sequence was also incorporated by connecting the CAR construct with 2A self‐cleavable linker sequence (Figure 1A), by which a suicide system can be triggered by exposure to GCV. We further designed an anti‐GM2 CAR construct with encoding human IL‐7 and CCL19 sequences, together with HSV‐TK (hereafter referred to as 7 × 19 CAR), as we have reported the significant improvement of therapeutic efficacy of CAR‐T cells in solid cancers by co‐expressing IL‐7 and CCL19.^9^, ^10^ When human PBMC were transduced with the retroviral vector of either Conv. CAR or 7 × 19 CAR, the transduction efficiencies of CAR were 89 and 85%, respectively (Figure 1B). Human IL‐7 and CCL19 were abundantly detected in the culture supernatant of 7 × 19 CAR‐T cells, but neither in those of Conv. CAR‐T cells nor un‐transduced (hereafter referred to as UTD) T cells (Figure 1C). Thus, these results confirmed that anti‐GM2 CAR constructs were successfully generated.

### 3.2 In vitro antitumor responses of anti‐GM2 CAR‐T cells

**FIGURE 2:**
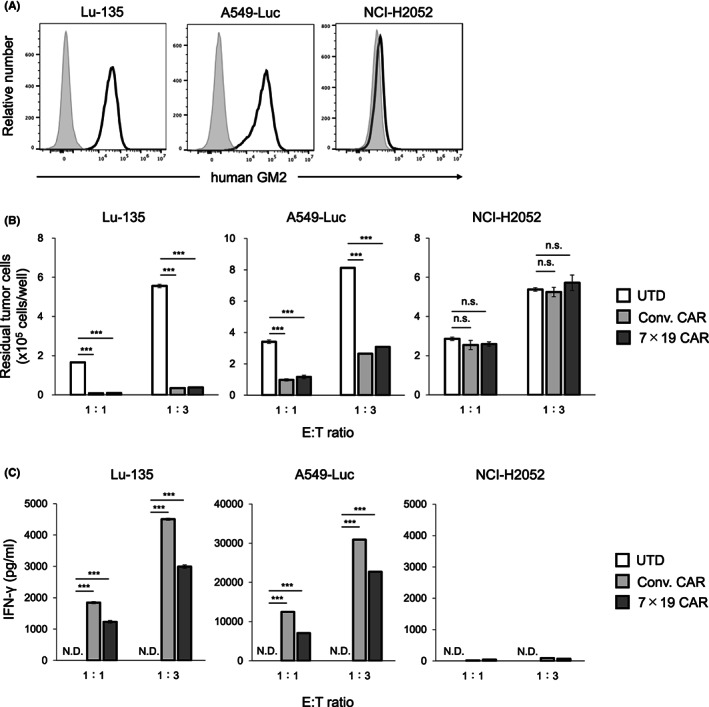
GM2‐specific immune responses of Conv. CAR and 7 × 19 CAR‐T cells in vitro. (A) Surface expression of endogenous GM2 was assessed in human solid cancer cell lines by flow cytometry. Open and filled histograms indicate staining with humanized anti‐GM2 Ab and nonstaining, followed by anti‐human IgG Ab, respectively. (B) Conv. CAR‐T, 7 × 19 CAR‐T, or UTD T cells were co‐cultured with the indicated tumor cells at an effector‐to‐target (E:T) ratio of 1:1 and 1:3 for 2 days. The number of residual tumor cells after co‐culture was analyzed by flow cytometry. Data are shown as mean ± standard error (SE) of triplicate samples. (C) The supernatants of co‐cultured cells as described in (B) were harvested, and the concentration of IFN‐γ was assessed by ELISA. Data are shown as mean ± SE of triplicate samples. ***p < 0.001, N.D., not detected; n.s., not significant.

We next evaluated the functions of Conv. and 7 × 19 CAR‐T cells in vitro in response to various human tumor cell lines with or without endogenous expressions of GM2. As GM2‐positive tumors, we used Lu‐135, a human SCLC cell line, and A549‐Luc, a human lung adenocarcinoma cell line (Figure 2A). As a GM2‐negative tumor, NCI‐H2052, a human mesothelioma cell line, was used. When GM2‐positive Lu‐135 or A549‐Luc tumors were co‐cultured with Conv. or 7 × 19 CAR‐T cells, the number of residual tumor cells was significantly reduced compared to that with UTD T cells (Figure 2B). On the other hand, Conv. and 7 × 19 CAR‐T cells showed no killing activity in the GM2‐negative NCI‐H2052 tumor. In addition, IFN‐γ secretion from Conv. and 7 × 19 CAR‐T cells, but not UTD T cells, was detected in the co‐culture with GM2‐positive Lu‐135 or A549‐Luc tumors, while negligible IFN‐γ was detected in the co‐culture with NCI‐H2052 cells (Figure 2C). Although the IFN‐γ production level of 7 × 19 CAR‐T cells was lower than that of Conv. CAR‐T cells in this model, it is not necessarily due to the presence of IL‐7 and/or CCL19 since 7 × 19 CAR‐T cells could produce a comparable or even higher IFN‐γ when 7 × 19 CAR‐T cells generated from a different donor were co‐cultured with a different tumor (data not shown). These results confirmed that the anti‐GM2 CAR‐T cells can express specific immune responses to GM2 antigen on tumor cells.

### 3.3 In vivo therapeutic effects of anti‐GM2 7 × 19 CAR‐T cells in a solid human SCLC model

**FIGURE 3:**
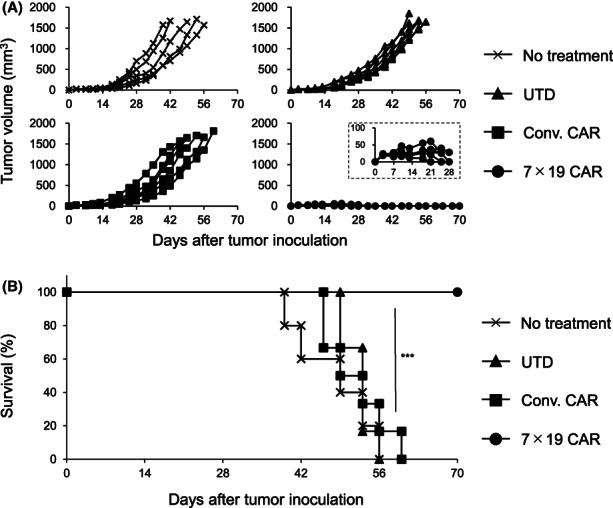
Potent therapeutic effects of 7 × 19 CAR‐T cells in the xenograft model of human small‐cell lung cancer. NOG‐ΔMHC mice were inoculated subcutaneously (s.c.) with 5 × 10^6^ Lu‐135 tumor cells on day 0 and then treated with intravenously injection of 1 × 10^7^ Conv. CAR‐T, 7 × 19 CAR‐T cells, or UTD T cells on day 3, or left untreated. Thereafter, the tumor size (A) and the mouse survival (B) were assessed (*n* = 5 in the untreated group, *n* = 6 each in UTD T cell, Conv. CAR‐T cell, and 7 × 19 CAR‐T cell‐treated groups). In (A), each line indicates the tumor volume of an individual mouse. Tumor volumes during the initial 28 days of 7 × 19 CAR‐T cell‐treated mice are shown in the inset. ***p < 0.001.

To investigate the antitumor effects of anti‐GM2 CAR‐T cells in vivo, we developed a pre‐established solid tumor model of human SCLC. Lu‐135 was inoculated s.c. into the flank of immunodeficient NOG‐ΔMHC mice on day 0, followed by i.v. administration with Conv. CAR‐T cells, 7 × 19 CAR‐T cells, or UTD T cells on day 3, or left untreated. Lu‐135 was completely rejected by the administration of 7 × 19 CAR‐T cells, but not the other groups (Figure 3A). Consistently, the survival of tumor‐inoculated mice was significantly extended by the treatment with 7 × 19 CAR‐T cells, but not the other groups (Figure 3B). Neurological toxicity such as head tilt, gait disturbance, and seizure was not observed in any of the treated mice (data not shown). These results indicated that 7 × 19 CAR‐T cells, but not Conv. CAR‐T cells, could induce potent therapeutic effects in the xenograft solid tumor model of human SCLC, although both CAR‐T cells showed a similar in vitro cytotoxic activity against GM2‐positive tumors as shown in Figure 2B.

### 3.4 Massive T cell infiltration in the tumor tissues of the mice treated with 7 × 19 CAR‐T cells

**FIGURE 4:**
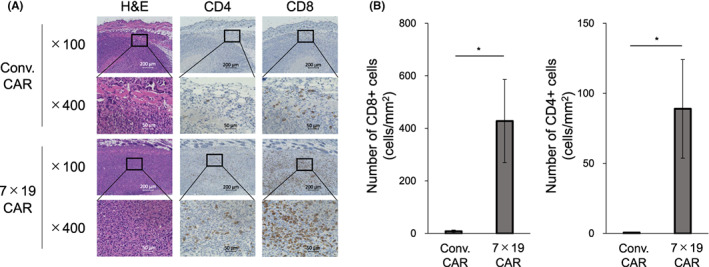
Massive infiltration of T cells in the tumor tissues by the treatment with 7 × 19 CAR‐T cells. NOG‐ΔMHC mice were inoculated subcutaneously with 1 × 10^7^ Lu‐135 cells on day 0, followed by treatment with intravenously injection of 1 × 10^7^ Conv. CAR‐T or 7 × 19 CAR‐T cells on day 3. Tumor tissues were resected from the mice on day 12, and FFPE slices were prepared from each tumor tissue and stained with H&E and IHC. In IHC staining, rabbit anti‐human CD4 and rabbit anti‐human CD8 mAb were used. Stained cells were visualized and observed by microscopic examinations at ×100 and ×400 magnifications. (A) Representative images are displayed. Scale bar indicates a length of 200 μm (×100) or 50 μm (×400). (B) The number of stained cell per tumor area (mm^2^) was calculated by BZ‐X analyzer software. Data are shown as mean ± standard error (*n* = 6 each per group). *p < 0.05.

In order to explore the mechanism of the distinct therapeutic effect between Conv. CAR‐T cells and 7 × 19 CAR‐T cells in the xenograft human SCLC model, we examined the level of T cell infiltration in the tumor tissues, since our previous studies revealed that the therapeutic effects of 7 × 19 CAR‐T cells were associated with massive infiltration of T cells inside the tumor tissues in several tumor models.^9^, ^10^ Lu‐135 tumor tissues were resected from the mice treated with Conv. or 7 × 19 CAR‐T cells and analyzed for the presence of T cells by IHC staining. We found that infiltration of CD4‐positive and CD8‐positive human T cells were significantly increased in the tumor tissues of the mice treated with anti‐GM2 7 × 19 CAR‐T cells compared to those with Conv. CAR‐T cells, whereas CD8‐positive T cells showed more prominent infiltration than CD4‐positive T cells (Figure 4A,B). We also found that infiltration of granzyme B‐positive cells was increased in the tumor tissues of the mice treated with anti‐GM2 7 × 19 CAR‐T cells compared to those with Conv. CAR‐T cells and almost no PD‐1‐positive cells were found in either group (Figure S1). These results indicated that simultaneous expressions of IL‐7 and CCL19 enhance T cell infiltration when applied to CAR‐T cells targeting GM2 tumor antigen, and those infiltrating T cells showed a cytolytic, but not exhausted, phenotype.

### 3.5 GM2‐specific memory responses induced by the treatment with 7 × 19 CAR‐T cells

**FIGURE 5:**
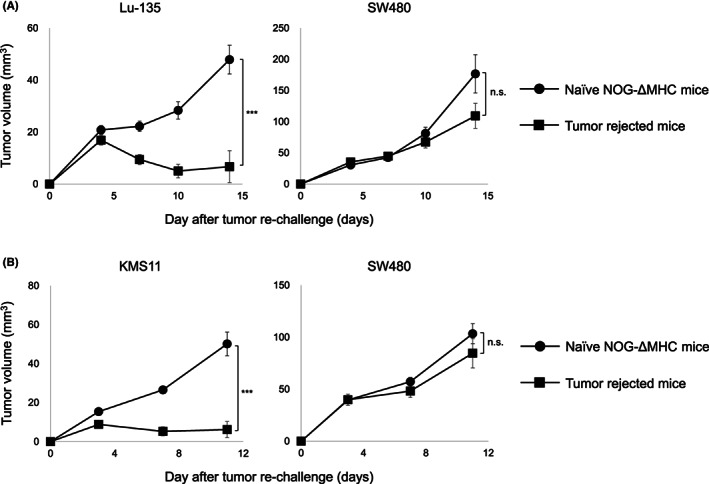
Generation of target‐specific long‐term memory responses by the treatment with 7 × 19 CAR‐T cells. NOG‐ΔMHC mice were inoculated subcutaneously (s.c.) with 1 × 10^7^ Lu‐135 cells on day 0, followed by treatment with intravenously injection of 1 × 10^7^ 7 × 19 CAR‐T cells on day 3. (A) The mice with complete tumor regression were rechallenged s.c. with 1 × 10^7^ Lu‐135 tumor cells at the right flank and 5 × 10^6^ SW480 tumor cells at the left flank on day 28. As a control, naïve NOG‐ΔMHC mice were inoculated s.c. with Lu‐135 and SW480 tumor cells in the same fashion (*n* = 6 in the tumor‐rejected mice group, *n* = 4 in the naïve NOG‐ΔMHC mice group). (B) The mice with a complete tumor regression were rechallenged s.c. with 3 × 10^6^ KMS11 tumor cells at the right flank and 5 × 10^6^ SW480 tumor cells at the left flank on day 35. As a control, naïve NOG‐ΔMHC mice were also inoculated s.c. with KMS11 and SW480 tumor cells in the same fashion (*n* = 4 in the tumor‐rejected mice group, *n* = 5 in the naïve NOG‐ΔMHC mice group). Tumor size was measured twice a week by a digital caliper. Tumor volumes are shown as mean ± standard error. ***p < 0.001, n.s., not significant.

We previously reported that long‐term antitumor memory response was generated by the treatment with 7 × 19 CAR‐T cells in both mouse and human tumor models.^9^, ^10^ Therefore, we next examined whether anti‐GM2 7 × 19 CAR‐T cells are also capable of inducing memory responses specific to GM2‐positive tumors. NOG‐ΔMHC mice that had achieved the complete rejection of Lu‐135 by the treatment with anti‐GM2 7 × 19 CAR‐T cells therapy were maintained for over 4 weeks, and then rechallenged with GM2‐positive or negative tumors. As a control, naïve NOG‐ΔMHC mice were also challenged with tumors in the same fashion. The growth of Lu‐135 in the tumor‐rejected mice was significantly inhibited compared to naïve mice, while the growth of GM2‐negative SW480 human colon tumor cells was comparable between these mice (Figure 5A). To further confirm a target specificity of the memory response, a separate set of the mice which had rejected Lu‐135 were rechallenged with KMS11, a human multiple myeloma cell line expressing GM2 (Figure S2). Tumor‐rejected mice significantly inhibited the growth of KMS11, but not SW480 (Figure 5B), suggesting that the long‐term memory responses induced by the treatment with anti‐GM2 7 × 19 CAR‐T cells were specific to GM2 antigen. Along with these observations, approximately 50% of CD8‐positive T cells in the tumor‐rejected mice showed CCR7‐positive, CD45RA‐positive stem cell memory phenotype (data not shown).

### 3.6 Depletion of HSV‐TK‐expressing 7 × 19 CAR‐T cells by GCV

**FIGURE 6:**
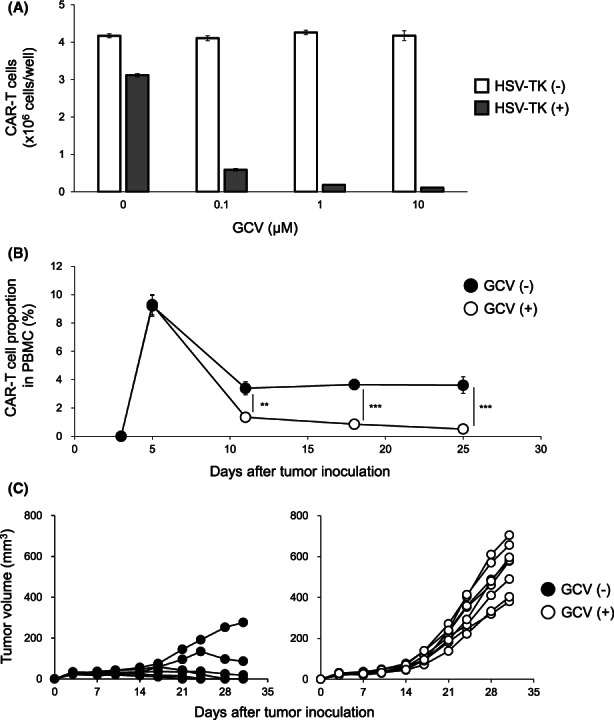
Depletion of 7 × 19 CAR‐T cells equipped with Herpes simplex virus‐thymidine kinase (HSV‐TK) by GCV treatment. (A) Anti‐GM2 7 × 19 CAR‐T cells equipped with or without HSV‐TK suicide gene were cultured for 3 days in the presence of GCV at a concentration of 0, 0.1, 1, and 10 μM. The number of residual CAR‐T cells was analyzed by flow cytometry. Data are shown as mean ± standard error (SE) of triplicate samples. (B, C) NOG‐ΔMHC mice were inoculated subcutaneously with 1 × 10^7^ Lu‐135 tumor cells on day 0, followed by intravenously injection of 1 × 10^7^ 7 × 19 CAR‐T cells expressing HSV‐TK on day 3, and then treated with i.p. administration of GCV (100 mg/kg) on days 7 and 21. (B) The proportion of CAR‐T cells in PBMC was assessed by flow cytometry. Data are shown as mean ± SE (*n* = 8 in the GCV‐treated mice group, *n* = 7 in nontreated mice group). **p < 0.01, ***p < 0.001. (C) Tumor size of Lu‐135 in the mice treated with 7 × 19 CAR‐T cells with or without GCV administration was measured twice a week by a digital caliper. Each line indicates the tumor volume of an individual mouse.

Anti‐GM2 7 × 19 CAR‐T cells generated in this study are equipped with HSV‐TK suicide gene, so as to eliminate CAR‐T cells if necessary. In order to confirm the function of HSV‐TK, effects of GCV were examined in vitro and in vivo. First, anti‐GM2 7 × 19 CAR‐T cells with or without HSV‐TK expression were cultured in the medium containing GCV at a concentration of 0, 0.1, 1, and 10 μM for 3 days. The number of residual CAR‐T cells was significantly decreased in a GCV dose‐dependent manner in 7 × 19 CAR‐T cells equipped with HSV‐TK, but not those without HSV‐TK (Figure 6A). Next, to evaluate the effects of HSV‐TK in vivo, NOG‐ΔMHC mice were inoculated s.c. with Lu‐135 on day 0, followed by i.v. administration of anti‐GM2 7 × 19 CAR‐T cells expressing HSV‐TK on day 3, and then treated with or without i.p. injections of GCV at 100 mg/kg on days 7 and 21. The percentage of CAR‐T cells in PBMC of the GCV‐treated mice was significantly decreased compared to non‐GCV‐treated mice (Figure 6B). In addition, the effects of 7 × 19 CAR‐T cells to inhibit the growth of Lu‐135 were attenuated by the GCV treatment (Figure 6C). These results demonstrated a susceptibility of anti‐GM2 7 × 19 CAR‐T cells equipped with HSV‐TK to GCV treatment, which enables to control of the immunological functions of 7 × 19 CAR‐T cells in vivo.

## 4 DISCUSSION

In this study, we revealed a potent therapeutic efficacy of anti‐GM2 7 × 19 CAR‐T cells against GM2‐positive human SCLC inoculated s.c. in immunodeficient mice, which were resistant to conventional anti‐GM2 CAR‐T cells. The therapeutic effects were associated with massive infiltration of T cells in tumor tissues and long‐term GM2‐specific memory responses to protect tumor regrowth. To the best of our knowledge, this study is the first report demonstrating GM2 as a target of CAR‐T cell therapy. In addition, this study supports the potential of the IL‐7/CCL19‐producing system as a platform technology applicable to various CAR‐T cells against solid cancers.

Our previous studies indicated that gene modification to express IL‐7 and CCL19 in antitumor effector T cells, such as CAR‐T and TCR‐T cells, profoundly enhances therapeutic effects for solid cancers by inducing a massive infiltration of immune cells in tumor tissues and long‐term memory responses.^9^, ^10^, ^11^ The results observed in the current study using anti‐GM2 CAR‐T cells are consistent with those previous findings. As this study adopted the model in which the injection of CAR‐T cells was performed at the early stage of tumor growth such as day 3, it is necessary and important to investigate the therapeutic efficacy of anti‐GM2 7 × 19 CAR‐T cells at the later stage of tumor development in future studies. GM2 is overexpressed in various cancer cells, while detected as a minor component in normal tissues including nervous and nonnervous cells.^12^, ^13^, ^14^, ^15^, ^16^, ^17^ The upregulated expression of GM2 in tumor cells has been shown to correlate with their malignant phenotypes, such as invasiveness of radiation‐tolerant lung cancer cells and resistance to chemotherapeutic drugs.^23^, ^24^ It was also reported that GM2 on tumor cells can mediate immunosuppressive effects through T cell dysfunction and apoptosis.^25^ Based on these biological features, GM2 has been investigated as a target of various immunotherapies, including anti‐GM2 mAb to mediate ADCC and GM2‐based vaccines to stimulate antitumor immune responses.^16^, ^17^, ^26^ Clinical trials of these approaches in patients of lung cancer, multiple myeloma, and melanoma showed no safety concerns, but its clinical efficacy was insufficient.^18^, ^26^, ^27^ These results suggested that more potent and durable immunotherapies targeting GM2 are necessary and thus motivated us to develop and examine the IL‐7/CCL19‐producing anti‐GM2 CAR‐T cells.

Besides GM2, several gangliosides including GD2, GD3, GM3, and fucosyl‐GM1 are also known to be overexpressed on cancer cells and associated, at least in part, with the malignant properties of cancers.^28^ Anticancer drugs targeting the gangliosides have been explored, in which anti‐GD2 CAR‐T cells are among the most advanced immune cell therapy. Administrations of anti‐GD2 CAR‐T cells in patients with neuroblastoma or glioma achieved clinical and radiographic responses in some cases without showing dose‐limiting toxicity.^19^, ^20^ Thus, CAR‐T cell therapy against gangliosides overexpressed on cancer cells is a promising approach, while neurological toxicity would be a crucial concern of the therapy since the gangliosides are expressed on central and peripheral nervous tissues, ranging from high to low levels. In a preclinical xenograft model of neuroblastoma, administration of 3 × 10^6^ CAR‐T cells expressing an affinity‐enhanced anti‐GD2 scFv has been shown to induce neurological toxicity with fatal encephalitis.^29^ In our current study, administration of 1 × 10^7^ anti‐GM2 CAR‐T cells expressing IL‐7/CCL19 demonstrated no symptoms associated with neurological toxicity, further supporting the clinical applicability of our approach. Although exact mechanisms of how CAR‐T cells targeting ganglioside distinguish between antitumor cytotoxicity and on‐target off‐tumor toxicity in nervous tissues remain unclear, it could be attributed to differences in the expression intensity and conformation of gangliosides on cell membrane between tumor cells and normal cells, as exemplified in GM3 and GM1.^30^, ^31^

One of the important clinical issues with CAR‐T cell therapy is how to control cytokine‐release syndrome (CRS) and immune effector cell‐associated neurotoxicity syndrome (ICANS), both of which are serious adverse events caused by excess activation of CAR‐T cells.^32^, ^33^ In addition, on‐target off‐tumor toxicities are also one of the important adverse events to overcome.^32^, ^33^ While treatment strategies for CRS, including a scoring system to evaluate clinical severity and an administration of tocilizumab, are being established, further approaches to control CAR‐T cell functions in vivo are required for better control of ICANS and on‐target off‐tumor toxicities. For this purpose, expressions of suicide genes or epitope sequences recognized by depletion mAb have been proposed as a method to selectively eliminate CAR‐T cells in vivo.^34^, ^35^, ^36^, ^37^ In this study, we generated anti‐GM2 7 × 19 CAR‐T cells expressing HSV‐TK and confirmed that GCV administration efficiently removes CAR‐T cells and attenuates their functions in vivo. Based on the previous studies in which the pharmacokinetics of GCV in mice and humans were examined,^38^, ^39^ the plasma concentration of GCV when injected in the mice with doses used in this study is expected to be lower than that of humans who are treated with a regular dose of GCV in the clinical setting, suggesting that our results in mouse model can be extrapolated to human. Thus, installment of HSV‐TK suicide system would be a useful approach to improve the clinical safety of 7 × 19 CAR‐T cell therapy.

In summary, this study revealed that GM2 can be a promising target in our platform CAR‐T cell technology expressing IL‐7 and CCL19 for the treatment of GM2‐positive solid cancers. Further development of anti‐GM2 7 × 19 CAR‐T cells and investigation of its efficacy and safety in clinical trials with solid cancer patients would be highly important and demanded in order to establish the next‐generation immunotherapy.

## AUTHOR CONTRIBUTIONS

**Takahiro Sasaki:** Data curation (lead); formal analysis (lead); investigation (lead); methodology (lead); writing – original draft (lead). **Yukimi Sakoda:** Conceptualization (supporting); methodology (supporting); project administration (supporting); supervision (supporting). **Keishi Adachi:** Conceptualization (supporting); methodology (supporting); supervision (supporting). **Yoshihiro Tokunaga:** Investigation (supporting); methodology (supporting); supervision (supporting). **Koji Tamada:** Conceptualization (lead); funding acquisition (lead); project administration (lead); supervision (lead); writing – review and editing (lead).

## CONFLICT OF INTEREST STATEMENT

Koji Tamada and Yukimi Sakoda hold stocks of Noile‐Immune Biotech and receive remuneration from Noile‐Immune Biotech. Koji Tamada received lecture fees from Ono Pharmaceutical, MSD, and Chugai Pharmaceutical. Koji Tamada received a research fund from Chugai Pharmaceutical. Yukimi Sakoda received a research fund from Noile‐Immune Biotech. Other authors declare no conflict of interest.

## Supporting information


Appendix S1.

## Data Availability

The data that support the findings of this study are available from the corresponding author upon reasonable request.
