# Supplementary material for: Therapeutic effects of anti‐GM2 CAR‐T cells expressing IL‐7 and CCL19 for GM2‐positive solid cancer in xenograft model
Source: Cancer Med. 2023 Apr 9;12(11):12569–80. doi: 10.1002/cam4.5907 (PMC10278466; doi:10.1002/cam4.5907)
Supplement: Supplementary file 1 — Appendix S1. [file CAM4-12-12569-s001.pdf]

Supplementary Figure 1

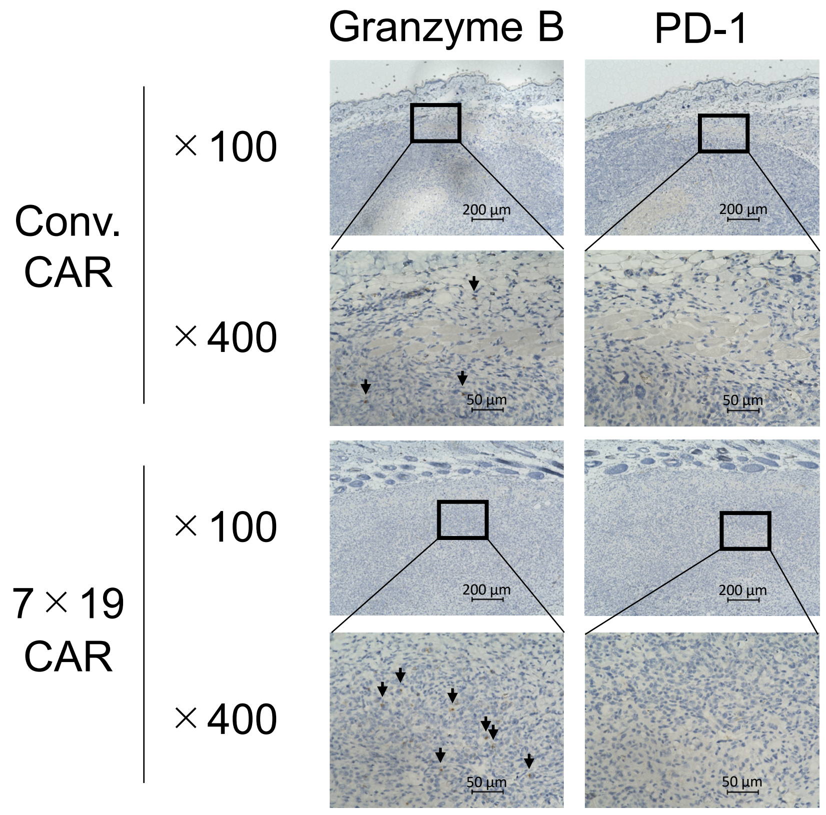

Supplementary Figure 1. Expression of Granzyme B and PD-1 on T cells in tumor tissues. NOG-ΔMHC mice were inoculated s.c. with 1×10^7 Lu-135 cells on day 0, followed by treatment with i.v. injection of 1×10^7 Conv. CAR-T or 7×19 CAR-T cells on day 3. Tumor tissues were resected from the mice on day 12, and FFPE slices were prepared from each tumor tissue and stained with IHC. In IHC staining, rabbit anti-human Granzyme B polyclonal Ab and mouse anti-human PD-1 mAb were used. Stained cells were visualized and observed by microscopic examinations at ×100 and ×400 magnifications. Representative images are displayed. Arrow indicates Granzyme B-positive cells. Scale bar indicates a length of 200 µm (×100) or 50 µm (×400).

Supplementary Figure 2

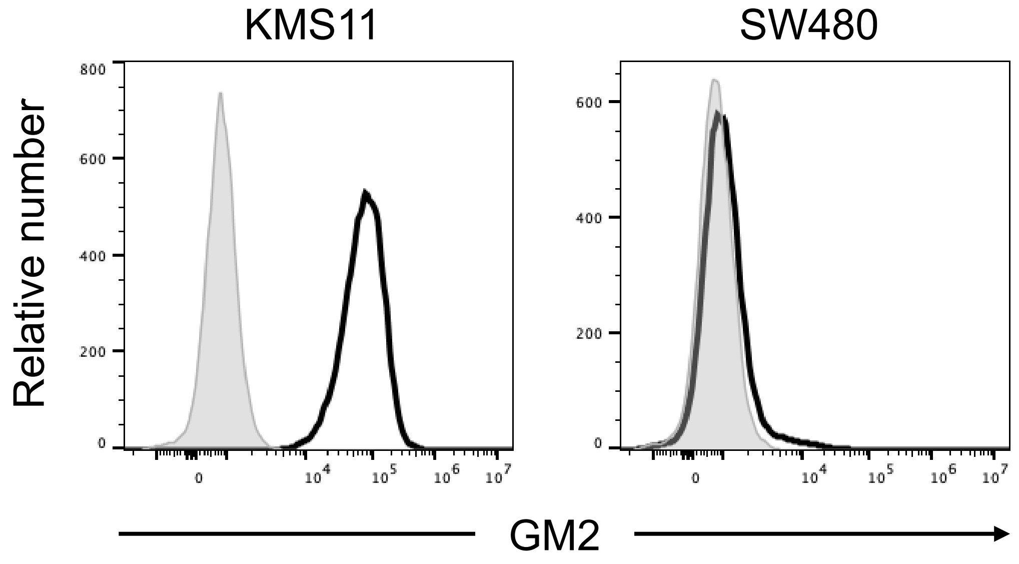

Supplementary Figure 2. Surface expression of GM2 on KMS11 and SW480.
Expression levels of GM2 on KMS11, a human multiple myeloma cell line, and SW480, a human colon tumor cell line, were assessed by flow cytometry. Open and filled histograms indicate staining with humanized anti-GM2 Ab and non-staining, followed by anti-human IgG Ab, respectively.
